# 胸壁外敷护理干预在达芬奇机器人肺癌手术后的应用效果

**DOI:** 10.3779/j.issn.1009-3419.2020.101.07

**Published:** 2020-06-20

**Authors:** 莺 王, 迪 孟, 新星 孙, 嘉琪 陶

**Affiliations:** 310003 杭州，浙江大学医学院附属第一医院 The First Affiliated Hospital of Zhejiang University, Hangzhou 310003, China

**Keywords:** 达芬奇机器人, 肺肿瘤, 护理干预, 芒硝, Da Vinci Surgical Robotic System, Lung neoplasms, Nursing interventions, Mirabilite

## Abstract

**背景与目的:**

微创与快速康复是肺癌外科治疗的趋势，而达芬奇机器人在肺癌的治疗及快速康复中起到重要作用。本研究探索在其基础上结合胸壁外敷的护理干预措施，对减少术后胸腔引流量、促进患者快速康复的作用。

**方法:**

对我院2017年11月1日-2018年4月2日行达芬奇机器人肺癌根治术的患者进行随机分组，对照组常规行达芬奇机器人肺癌根治术，术后胸部缠绕腹带。实验组行达芬奇机器人肺癌根治术，术后辅助胸壁局部外敷芒硝进行护理干预。统计相关数据并分析。

**结果:**

实验组的术后总引流量、日均引流量均少于对照组，术后拔管时间短于对照组，住院时间较对照组缩短，但无明显统计学差异。术后第2天疼痛评分上，实验组略高于对照组，无明显统计学差异。对于胸壁厚度≤4 cm的患者，胸壁外敷的护理干预可显著减少术后日均及总引流量，但在拔管时间、住院时间上无明显差异。

**结论:**

胸壁外敷护理干预有利于达芬奇机器人肺癌手术患者的术后恢复，尤其对于胸壁厚度较小的患者，能够减少术后引流量，缩短留管时间，加快出院。进一步改良后有望达到更好的临床效果。

## Abstract (en)

**Background and objective:**

Minimally invasive and rapid recovery are trends in surgical treatment of lung cancer, and Da Vinci Surgical Robotic System plays an important role in them. This study was planned to explore the effect of chest wall external nursing interventions on reducing postoperative thoracic drainage and promoting rapid recovery of patients.

**Methods:**

The patients who underwent robotic radical lung cancer resection in our hospital from November 2017 to April 2018were randomly divided into two groups. The control group received robotic radical lung cancer resection with abdominal bands wrapped around the chest. The experimental group underwent Da Vinci robotic radical lung cancer surgery and assisted chest wall external nursing interventions after surgery.

**Results:**

The total and average daily drainage of the experimental group were less than those of the control group. Both the extubation time and the hospitalization time were shorter than those of the control group, but there was no significant difference. The pain score in the second days after operation of the experimental group was slightly higher than that of the control group, with no significant statistical difference. For patients whose chest wall thickness is less than 4 cm, mirabilite external application can significantly reduce the average daily and total drainage volume, but there is no significant difference in extubation time and hospitalization time.

**Conclusion:**

Chest wall external nursing interventions are beneficial to the recovery of patients undergoing Vinci robotic lung cancer surgery. Especially for patients with thinner chest wall. It can reduce postoperative drainage, shorten tube retention time and accelerate discharge. Further improvement is expected to achieve better clinical results.

肺癌是目前世界范围内的第一大癌症，发病率及死亡率均位居首位。外科手术切除是治疗肺癌的重要手段。而达芬奇机器人手术系统由于其高分辨率的三维立体视野、颤抖过滤、高自由度关节等优势，在肺癌手术中应用广泛。随着手术技术的不断发展和对疾病认识的不断深入，手术后快速康复（enhanced recovery after surgery, ERAS）理念深入人心，而围术期护理干预的优化是其重要内容之一。肺癌术后的拔管时间决定了患者术后住院时间，而影响拔管的因素中，术后胸腔引流量至关重要。

祖国医学认为，胸腔积液属于悬饮范畴，其病机为“阳虚阴盛，水饮内停”，治疗上以“温阳利水”为其大法。芒硝作为常用的传统中药，其内服有泻下作用，外敷则起到收敛、消肿作用。芒硝为矿物类中药，分布广泛，其主要成份是硫酸钠，性味苦、咸、寒，归胃、大肠经。20世纪50年代以来医药界专家对芒硝研究逐渐增多，并取得丰硕成果，研究表明芒硝具有软坚散结、清热解毒、抗炎、吸湿消肿等作用。2010版《中国药典》记载其功能为泻下通便、润燥软坚、清火消肿，用于实热积滞、腹满胀痛、大便燥结、肠痈肿痛；外治乳痈、痔疮肿痛。芒硝临床应用以外敷为主，一般不入煎剂，外敷治疗丹毒、静脉炎、结石、乳痈等疾病效果显著。本研究旨在开展达芬奇机器人手术的基础上，探究局部胸壁芒硝外敷的护理干预手段对于减少肺癌患者术后引流量、加快康复出院的效果。

## 1 资料与方法

### 1.1 一般资料

随机抽取我院2017年11月1日-2018年4月2日行达芬奇机器人肺癌根治术的患者作为临床资料进行统计分析，纳入条件：①行达芬奇机器人肺癌根治手术（肺叶/楔形切除+淋巴结清扫术）；②术后病理证实为肺癌。排除标准：①不符合上述标准的患者；②术中发现中度及以上胸膜粘连的患者；③术后出现出血、漏气、乳糜胸等并发症影响拔管的患者。

### 1.2 治疗方法

患者随机分为两组，住院号尾数为单数者为对照组，双数者为实验组。对照组常规行达芬奇机器人肺癌根治术，术后胸部缠绕腹带。实验组行达芬奇机器人肺癌根治术，术后由巡回护士将1 kg芒硝置于30 cm×20 cm的双层棉布袋中，以腹带固定于患者术侧胸腔。术后持续外敷直至出院。

### 1.3 观察指标

统计患者的性别、年龄、身体质量指数（body mass index, BMI）、手术部位、病理类型等临床资料。通过术前肺部CT测量手术侧胸壁厚度（腋中线第7肋间）。患者术后的观察指标包括：每日引流量、术后总引流量、每日疼痛评分、术后拔管时间、术后住院时间。

### 1.4 统计方法

所有数据通过SPSS软件计算分析，根据数据性质分别采用均数或非参数等级资料比较，计算各组统计学差异。*P* < 0.05表示差异有统计学意义。

## 2 结果

### 2.1 一般资料

纳入的研究对象均为本院达芬奇机器人肺癌手术患者，手术主刀医生为同一位主任医师。研究共纳入我院2017年11月1日-2018年4月2日行达芬奇机器人肺癌根治术的患者100例，对照组及实验组各50例。两组患者的临床特征见表 1，结果显示，两组研究对象在性别、年龄、手术类型、BMI及胸壁厚度上均无明显差异，病理类型上均以腺癌为主。

**1:** 患者的临床特征 Clinical characteristics of patients

	Control group (*n*=50)	Experimental group (*n*=50)	*P*
Gender			
Male	18	20	0.32
Female	32	30	
Age	46.7	42.5	0.63
Surgery method			
Wedge resection	21	27	0.76
Lobectomy	29	23	
Body mass index (BMI, kg/m^2^)	21.37	23.58	0.21
Chest wall thickness (cm)	4.21	4.75	0.48

### 2.2 观察指标

统计结果显示（表 2），实验组的术后总引流量、日均引流量均少于对照组，术后拔管时间短于对照组，住院时间较对照组缩短，但无明显统计学差异。术后第2天疼痛评分上，实验组略高于对照组，无明显统计学差异。考虑到芒硝外敷对胸腔积液的收敛效果可能受到胸壁厚度的影响，我们根据不同的胸壁厚度进行分类比较。结果显示，胸壁厚度是影响芒硝外敷效果的重要因素，当胸壁厚度≤4 cm时，芒硝外敷可显著减少术后日均及总引流量，但在拔管时间、住院时间上无明显差异（表 3）。术后第2天疼痛评分上，实验组仍略高于对照组，无明显统计学差异。

**2:** 两组患者术后相关资料比较（Mean±SD） The postoperative clinical data between the two groups (Mean±SD)

	Control group (*n*=50)	Experimental group (*n*=50)	*P*
Total chest drainage (mL)	771.60±32.40	724.80±43.20	0.332
Daily chest drainage (mL)	253.82±12.47	244.86±14.32	0.521
Duration of chest drainage (d)	3.04±1.03	2.96±1.12	0.173
Post-operative hospital day (d)	4.24±0.95	4.12±1.04	0.253
Post-operative VAS score on day 2	2.34±1.27	2.74±1.14	0.43
VAS: visual analogue scale			

**3:** 两组患者术后相关资料比较（胸壁厚度≤4 cm）（Mean±SD） The postoperative clinical data between the two groups (chest wall thickness ≤4 cm)(Mean±SD)

	Control group (*n*=50)	Experimental group (*n*=50)	*P*
Total chest drainage (mL)	730.83±33.53	685.16±38.52	0.042
Daily chest drainage (mL)	261.95±12.41	250.06±13.64	0.07
Duration of chest drainage (d)	2.79±0.94	2.74±0.96	0.57
Post-operative hospital day (d)	2.92±0.92	2.93±0.92	0.61
Post-operative VAS score on day 2	2.42±1.24	2.55±1.36	0.77

## 3 讨论

近年来，随着微创外科技术的飞速进步以及ERAS的全面发展，肺癌的手术治疗已经从传统的开放手术发展到目前的微创外科手术，其中达芬奇机器人手术系统更是打开了微创手术发展的新篇章。自2002年Mariani等^[1]^报道了全球首例应用达芬奇机器人手术系统行肺癌根治术以来，经过多年的临床研究与验证，达芬奇机器人行肺癌根治术已得到广泛认可^[2, 3]^。我院自2014年12月引进达芬奇机器人手术系统，截止2019年7月，已完成各类胸外科手术1, 000余例，无重大并发症，患者均得到了良好的手术效果和快速的术后康复。

ERAS理念涵盖了外科微创手术、麻醉方式和围术期护理干预等三方面^[4]^。在临床实践中，围术期的护理干预是ERAS不可或缺的一环，有很大的应用前景。多项研究表明，围术期护理干预可以有效减少患者住院时间、医疗费用，促进患者康复^[5-7]^。在享受高新科技和ERAS理念带来的医学进步的同时，我们也不断探索祖国医学中的宝贵财富。芒硝的主要成分是含水硫酸钠，主要功效是泻下、软坚、清热。芒硝外敷能改善血液循环，使网状内皮系统吞噬功能加强，从而调动机体免疫力^[8]^；此外，还能大量摄取体表渗出液^[9]^。在2010版《中国药典》中，记载其功能为泻下通便、润燥软坚、清火消肿，用于实热积滞、腹满胀痛、大便燥结、肠痈肿痛；外治乳痈、痔疮肿痛。在临床实践中，芒硝外敷用于重症胰腺炎、肝硬化性腹水、肝癌腹水等疾病中均取得了显著的疗效^[10, 11]^，不仅能够减少腹水的产生，还能缓解腹胀、促进肠动力恢复^[12, 13]^。在输液后常见的静脉炎的治疗上，芒硝外敷可以降低白介素、肿瘤坏死因子（tumor necrosis factor, TNF）-α等炎症因子表达水平，减轻炎症反应，从而改善静脉炎症与疼痛^[14, 15]^。在本研究中，我们创新性地首次将芒硝应用于胸腔积液的治疗，也取得了一定的疗效。我们的研究结果显示，行达芬奇机器人肺癌手术的患者，术侧胸腔接受芒硝外敷，可减少术后引流量，缩短术后留管时间，加快患者康复出院，证明芒硝在胸腔积液的治疗上也可起到一定作用。

此外我们发现，胸壁厚度是影响芒硝收敛作用的一个重要因素。在腹部的治疗应用中，芒硝主要外敷于脐周。脐部是人体胚胎发育过程中腹壁最后闭合处，表皮角质层最薄，屏障功能最弱，有利于腹水的析出^[16]^。由于患者胸壁厚度不同，芒硝的作用效果也不尽相同。胸壁的厚度主要取决于患者皮下脂肪层的厚度，对于BMI较高、胸壁脂肪较多，尤其是胸壁厚度 > 4 cm的患者，芒硝外敷的作用有限。

在本研究的护理干预过程中，我们也发现了一些芒硝外敷的问题。首先，芒硝外敷的技巧仍需完善。在本研究中，我们将1 kg芒硝置于30 cm×20 cm的双层棉布袋中，以腹带固定于患者术侧胸腔。部分患者由于布袋卡压胸腔引流管，引起管口疼痛，影响术后休息。且芒硝吸水后结块变硬，腹带挤压固定后易引起患者不适甚至疼痛感。此外，由于ERAS流程要求患者术后第2天下床活动，在坐位及直立体位时，芒硝布袋容易掉落难以固定，外敷时间无法充分保证。其次，芒硝的外用剂量尚无明确定量研究，如何平衡芒硝用量、更换频率与外敷效果、患者使用舒适度，仍需进一步研究。

总之，护理干预芒硝外敷有利于达芬奇机器人肺癌手术患者的术后恢复，尤其对于胸壁厚度较小的患者，能够减少术后引流量，缩短留管时间，加快出院。但是在具体使用过程中，尚需改良外敷技巧及用量，以达到更好的临床效果。
